# A spatiotemporal model to assess the introduction risk of African horse sickness by import of animals and vectors in France

**DOI:** 10.1186/s12917-015-0435-4

**Published:** 2015-06-04

**Authors:** C. Faverjon, A. Leblond, P. Hendrikx, T. Balenghien, C. J. de Vos, E.A.J. Fischer, A.A. de Koeijer

**Affiliations:** INRA UR346 Animal Epidemiology, Vetagrosup, F-69280 Marcy l’Etoile, France; INRA UR346 Animal Epidemiology et Département Hippique, VetAgroSup, F-69280 Marcy L’Etoile, France; ANSES, Direction scientifique des laboratoires – unité Survepi, 94700 Maisons-Alfort, France; CIRAD, UMR CMAEE, F-34398 Montpellier, France ; INRA, UMR1309 CMAEE, F-34398 Montpellier, France; Central Veterinary Institute, part of Wageningen UR, PO Box 65, 8200 AB Lelystad, The Netherlands

**Keywords:** African horse sickness, Equine movements, Import risk assessment, Risk of introduction, *Culicoides*, Quantitative risk, Midge

## Abstract

**Background:**

African horse sickness (AHS) is a major, *Culicoides*-borne viral disease in equines whose introduction into Europe could have dramatic consequences. The disease is considered to be endemic in sub-Saharan Africa. Recent introductions of other *Culicoides*-borne viruses (bluetongue and Schmallenberg) into northern Europe have highlighted the risk that AHS may arrive in Europe as well. The aim of our study was to provide a spatiotemporal quantitative risk model of AHS introduction into France. The study focused on two pathways of introduction: the arrival of an infectious host (PW-host) and the arrival of an infectious *Culicoides* midge via the livestock trade (PW-vector). The risk of introduction was calculated by determining the probability of an infectious animal or vector entering the country and the probability of the virus then becoming established: i.e., the virus’s arrival in France resulting in at least one local equine host being infected by one local vector. This risk was assessed using data from three consecutive years (2010 to 2012) for 22 regions in France.

**Results:**

The results of the model indicate that the annual risk of AHS being introduced to France is very low but that major spatiotemporal differences exist. For both introduction pathways, risk is higher from July to October and peaks in July. In general, regions with warmer climates are more at risk, as are colder regions with larger equine populations; however, regional variation in animal importation patterns (number and species) also play a major role in determining risk. Despite the low probability that AHSV is present in the EU, intra-EU trade of equines contributes most to the risk of AHSV introduction to France because it involves a large number of horse movements.

**Conclusion:**

It is important to address spatiotemporal differences when assessing the risk of ASH introduction and thus also when implementing efficient surveillance efforts. The methods and results of this study may help develop surveillance techniques and other risk reduction measures that will prevent the introduction of AHS or minimize AHS’ potential impact once introduced, both in France and the rest of Europe.

**Electronic supplementary material:**

The online version of this article (doi:10.1186/s12917-015-0435-4) contains supplementary material, which is available to authorized users.

## Background

African Horse Sickness (AHS) is a highly fatal viral vector-borne disease that is transmitted among equine hosts by *Culicoides* midges (Diptera: Ceratoponidae) [1, 2]. It affects all extant Equidae, but morbidity and mortality vary among species: as many as 90 % of infected horses die within one week, while infection is largely subclinical in zebras [3, 4]. AHS virus (AHSV) is an orbivirus, and there are nine different ASHV serotypes that confer some degree of cross-protective immunity [4]. AHS is considered to be endemic in sub-Saharan Africa, where the zebra acts as a reservoir [5]. Rare outbreaks have occurred in North Africa, western Asia, and the Iberian Peninsula, where they have persisted for only a few years [6]. The last outbreak in Europe occurred in the Iberian Peninsula, between 1987 and 1990, and caused the death of more than 1,350 horses, either directly or as a result of control measures [7].

The recent introduction into northern Europe of bluetongue virus (specifically BTV-8, in 2006 [8]) and Schmallenberg virus (in 2011 [9]), both transmitted by *Culicoides* midges, highlights the relevance of assessing the risk that AHSV will be introduced to Europe [10]. It is particularly crucial to conduct a risk assessment analysis for France, as the country encompasses different ecosystems, including Mediterranean zones, where *Culicoides imicola*—considered to be the major vector of AHSV worldwide—is very abundant, and non-Mediterranean temperate zones, where *Culicoides obsoletus—*a potential AHSV vector—is dominant [11, 1, 12]. Moreover, France contains between 900,000 and 1 million equines, is the world’s 4th largest exporter of horses, and has a horse industry that produces around 12 billion euros of revenue per year [13]. If AHS arrived in France, it could have devastating consequences, similar to those predicted for other EU members such as the United Kingdom (UK) [14], Ireland [15], and the Netherlands [16].

Introduction risks have recently been quantitatively assessed for similar vector-borne diseases, such as BTV [17–19], West Nile Virus [20, 21] and eastern and western equine encephalomyelitis [22], Venezuelan equine encephalitis [22], and Japanese encephalitis [22]. However, these studies mostly took into account only one pathway of introduction; different introduction pathways have rarely been examined in tandem. To explore AHSV in particular, a qualitative risk assessment analysis that accounted for multiple pathways of introduction was conducted in the UK [23]; the results suggested that the most likely pathway of introduction would be the arrival of an infectious host. This pathway of introduction was also examined by a quantitative risk assessment analysis of the likelihood that AHSV would be introduced to the Netherlands [24]. As AHSV is closely related to BTV and shares the same vectors, information on BTV introduction pathways could be helpful when assessing the risk that AHSV will be introduced to Europe. Several studies have indicated that long-distance, wind-mediated transport of *Culicoides* might have played a role in the introduction and spread of several BTV strains in Europe [25–27]. In particular, studies aimed at understanding the introduction of BTV-8 have indicated that the legal importation of an infectious host is unlikely to have caused the epidemic observed in 2006 [28, 29]. It is thought that the risk presented by other pathways of introduction, such as the introduction of a single *Culicoides* midge through intracontinental transport and trade networks [19], is low. Integrative studies are required to quantify and combine information on different pathways of introduction to better understand and confront the risks posed by vector-borne diseases [30].

In this study, we performed a quantitative risk assessment analysis of the introduction of AHSV to France. We focused on two pathways of introduction: the arrival of an infectious host and the arrival of an infectious *Culicoides* midge via the livestock trade. Introduction is defined here as the probability that an infectious host or vector will be released in such a way that at least one local host ends up infected by one local vector (establishment). The subsequent spread of the disease is not examined. As the initial infection of a local host will depend on spatial (e.g., the number of local hosts) and temporal factors (e.g., seasonal vector abundance), the probability of establishment will vary depending on location and time period. The objective of this study was to quantify the risk of introduction associated with a given time period and region for the two pathways of introduction under consideration, which could thus offer insight into temporal and regional variation in introduction risk. Furthermore, evaluating these two pathways of introduction could help optimize risk-mitigating control and surveillance measures.

## Methods

### Risk associated with introduction pathways and initial assumptions

To quantify the risk of AHSV introduction associated with the two introduction pathways, risk assessment analysis was conducted using the framework developed by de Vos et al. [31]. Although other potential pathways of introduction exist [23], we restricted ourselves to the two most probable: the arrival of an infectious equine and the arrival of an infectious *Culicoides* midge. Only the legal, registered horse trade was taken into account because no data exist on the illegal horse trade. In the analysis, only the introduction of an adult vector was considered since transovarial transmission of the virus has not been observed in *Culicoides* [1, 11, 15]. *Culicoides* midges are hematophagous and tend to stay close to their mammalian hosts (mainly large mammals [32, 33]). They are rarely found in vehicles of transport (such as aircraft or trucks) or merchandise when insect surveys are conducted [21, 34]. It is also uncommon to find *Culicoides* associated with plants or plant material [29, 35]. As a result, only *Culicoides* entering the country via the livestock trade were included in the analysis. Hence, the two main pathways of AHSV introduction examined in this study were: the legal importation of infectious equines (PW-host) and the arrival of infectious *Culicoides* as a consequence of livestock trade (PW-vector).

**Fig. 1:**
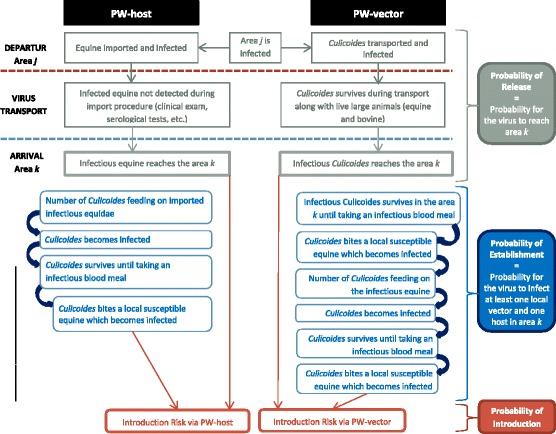
Introduction pathways. Steps required for the successful release and establishment of AHSV resulting in at least one local host infected by one local vector

An introduction pathway was constructed to detail all the steps required for AHSV to be successfully released and become established in France (Fig. 1). This introduction pathway was evaluated using a stochastic risk simulation model. Monthly introduction probabilities were calculated using data from three consecutive years (2010 to 2012) for each area of arrival within France. A total of 22 such areas were defined.

### Sources of risk

**Fig. 2:**
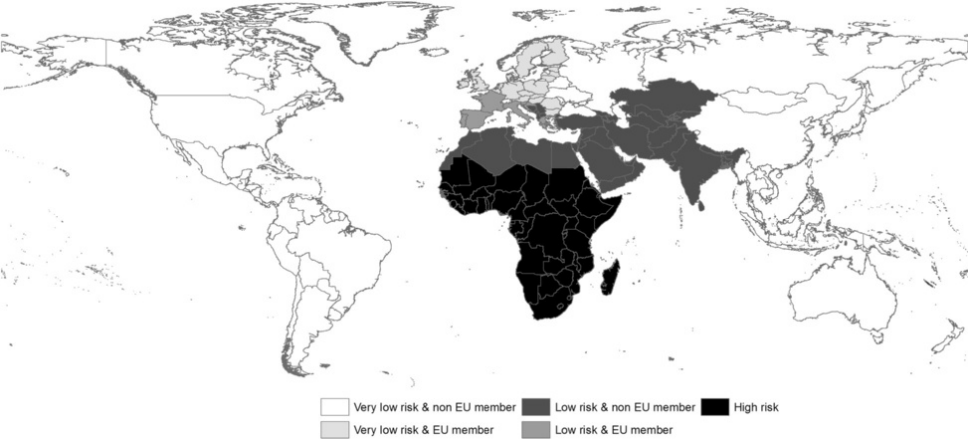
Countries of the world classified regarding AHS occurrence and import regulations

First, the world’s countries were grouped into three categories as per De Vos et al. [24]: (1) high-risk regions where the disease is considered to be endemic; (2) low-risk regions that have experienced AHS outbreaks in the past and/or where the main vector, *C. imicola*, is present; and (3) very-low-risk regions (all other countries). Since the main vector is not present in very-low-risk regions, we assumed that it is very unlikely that they would produce exports containing infectious vectors; consequently, very-low-risk regions were ignored for this pathway of introduction. In addition, because EU regulations differ for imports arriving from EU versus non-EU countries [36], we also distinguished between (a) EU members and (b) non-EU members. Five departure regions were thus defined (Fig. 2). Imports from non-EU countries were placed in one of two categories based on their point of arrival in Europe: whether they were shipped directly to France or whether they arrived via another EU country, because animals stopping in another country were considered to experience longer traveling times. Furthermore, equine imports were grouped according to species: (1) horses; (2) donkeys, mules, and hinnies; and (3) zebras.

### The model

#### PW-host: introduction via an infectious host

The probability of AHSV being introduced by species *i* from region *j* to area *k* in month *m* via an infectious host (PW-host), (*introH*_*ijkm*_), was defined as the probability of at least one infectious host of species *i* from region *j* arriving in area *k* in month *m* and of this arrival being followed by virus transmission to a local vector and host. This overall probability was defined as:

1 $$ P\left( intro{H}_{ijkm}\right)=1-{\left[1-P\left(rel{H}_{ijkm}\right)\times P\left(est{H}_{ijkm}\right)\right]}^{e{q}_{ijkm}} $$

Where *P*(*relH*_*ijkm*_) is the probability of an infectious equine of species *i* from region *j* being released in area *k* in month *m*; *P*(*estH*_*ijkm*_) is the probability of an infection becoming established in month *m* given the release of one infectious equine of species *i* from region *j* in region *k*; and *eq*_*ijkm*_ is the number of equines of species *i* imported from region *j* arriving in area *k* in month *m.*

Release probabilities, *P*(*relH*_*ijkm*_), were species specific since virus prevalence is different in different equines across the areas of origin, and different species show differences in their susceptibility to the disease. For instance, the release probability of horses is lower because horses have a shorter viremic period than do donkeys and zebras. *P*(*relH*_*ijkm*_) also depended on the moment *z* of infection; the protective measures implemented before embarkation [36, 37]; and the duration of transport from region *j* to area *k* (*t*_*jk*_). For imports coming from non-EU countries, *t*_*jk*_ was assumed to equal 1 day for animals coming directly to France (mainly air travel) and 2 days for animals arriving via another EU country (initial air travel followed by land transport or subsequent air travel). For intra-EU trade, *t*_*jk*_ was assumed to be between 1–2 days (uniform distribution) because air and land transport are supposed to be used with equal frequency and France is assumed to be a maximum of 2 days away from everywhere else in EU [38]. *P*(*relH*_*ijkm*_) was defined as :

2 $$ P\left(rel{H}_{ijkm}\right)=\frac{{\displaystyle {\sum}_{\mathrm{z}=1}^{\mathrm{w}}}\left[\left(\mathrm{length}\ \mathrm{period}\ \mathrm{z}\right)\times \mathrm{P}\left({\mathrm{relH}}_{\mathrm{ijkmz}}\right)\right]}{{\displaystyle {\sum}_{\mathrm{z}=1}^{\mathrm{w}}}\left(\mathrm{length}\ \mathrm{period}\ \mathrm{z}\right)} $$

where *P*(*relH*_*ijkmz*_) is the probability of release for an equine *i* infected during *z*. For a given region *j*, there were a total of *w* different time periods *z* during which an equine could become infected, depending on the importation procedures implemented for region *j* (e.g., before quarantine or during quarantine but before the first serological test, etc.). If *j* was a high-risk region, then *P*(*relH*_*ijkmz*_) was defined as:

2.bis $$ P\left(rel{H}_{ijkmz}\right)=P\left( in{f}_{ijmz}\right)\times P\left(vi{r}_{ijmz}\right)\times \left(1-P\left(CF{1}_{iz}\right)\right)\times \left(1-P\left(CF{2}_{iz}\right)\right)\times \left(1-P\left(cli{n}_{ijmz}\right)\right)\times \left(1-P\left( tran{s}_{ijkz}\right)\right) $$

Where *P*(*inf*_*ijmz*_) is the probability of equine *i* being infected during time period *z*; $P\left({vir}_{ijmz}\right)$ is the probability of equine *i* infected during *z* becoming viremic after transport; (1 − *P*(*CF*1_*iz*_)) and (1 − *P*(*CF*2_*iz*_)) are the probabilities of equine *i* infected during *z* not being detected by the first and second serological tests, respectively; (1 − *P*(*clin*_*ijmz*_)) is the probability of equine *i* infected during *z* not being detected by the clinical exam; and (1 − *P*(*trans*_*ijkz*_)) is the probability of equine *i* infected during *z* not being detected during transport.

The probability of establishment via PW-host was defined as:

3 $$ P\left(est{H}_{ijkm}\right)=1-{\left[1-{\lambda}_{HV}\times P\left(sur{v}_{km}\right)\times {b}_{equ{i}_k}\times {\lambda}_{VH}\right]}^{cul{i}_{km}} $$

where *λ*_*HV*_ and *λ*_*VH*_ are, respectively, the probabilities of a vector feeding on an infectious host becoming infected and of a host bitten by an infectious vector becoming infected; *P*(*surv*_*km*_) is the probability of an infected *Culicoides* midge surviving until its first infectious blood meal; *culi*_*km*_ is the number of vectors feeding on an infectious imported host; and ${b}_{equ{i}_k}$ is the probability of a *Culicoides* midge biting an equine in area *k*${b}_{equ{i}_k}$ depends on the vector’s preference for equines as hosts and on the cattle-to-equine ratio in area *k*.

The overall national and annual median probabilities were calculated based on the monthly regional values. The monthly national median probability of introduction was thus defined as:

4 $$ P\left( intro{H}_{ijm}\right)=1-{\displaystyle {\prod}_{K=1}^{22}\left(1-P\left( intro{H}_{ijkm}\right)\right)} $$

and the annual national median probability of introduction was defined as:

5 $$ P\left( intro{H}_{ij}\right)=1-{\displaystyle {\prod}_{m=1}^{12}\left(1-P\left( intro{H}_{ijm}\right)\right)} $$

The same formulas were used to define the probabilities of release and establishment at the national and annual levels. Using these formulas, the extreme values of these probabilities were determined by using the estimated the 5th and 95th percentiles for each region and month.

For a more detailed description of the probabilities and parameters used, see Additional files 1 and 2.

#### PW-vector: introduction via an infectious vector

Few data were found on the number of *Culicoides* midges transported with livestock (equines and cattle) over the distances of interest here; as a consequence, we assumed that the numbers could not be very high without having spurred notice and thus calculated the risk of release assuming that one *Culicoides* was transported with each animal. We defined the probability of establishment as the probability that this single vector was able to cause the infection of at least one local equine host by one local vector. *P*(*introV*_*jkm*_) was thus define as:

6 $$ P\left( intro{V}_{jkm}\right)=1-{\left[1-P\left(rel{V}_{jkm}\right)\times P\left(est{V}_{jkm}\right)\right]}^{{\mathrm{n}}_{\mathrm{jkm}}} $$

where *P*(*relV*_*jkm*_) is the probability of a single infected *Culicoides* from region *j* being released in region *k* in month *m*; *P*(*estV*_*jkm*_) is the probability of establishment; and *n*_*jkm*_ is the number of livestock (equines and bovines) transported from region *j* to area *k* during month *m*.

The probability of release was defined as:

7 $$ P\left(rel{V}_{jkm}\right)=P\left( inf\_cul{i}_{jm}\right)\times P\left( tran{s}_{cul{i}_{jm}}\right)\times P\left(sur{v_{trans}}_{jkm}\right) $$

where *P*(*inf*_*culi*_*jm*_) is the probability of a vector in region *j* becoming infected in month *m*; $P\left( tran{s}_{cul{i}_{jm}}\right)$ is the probability of a vector being transported post infection; and *P*(*surv*_*transjkm*_) is the probability of a *Culicoides* surviving transport from region *j* to region *k. P*(*surv*_*transjkm*_) was calculated assuming that transport conditions do not affect *Culicoides* viability (worst case scenario because the survival of insects is supposed to be optimal during transport). If pest control is implemented in region *j*, we reduced survival probabilities depending on the efficiency of the pest control product (*Prot*_*vect*_) used. This was only the case for equines coming from high-risk regions [36]. Since bovines are not consistently and systematically disinfected before transport, we assumed that no pest control was implemented for them. The probability of establishment via an infectied vector was defined as:

8 $$ P\left(est{V}_{jkm}\right)=P\left(sur{v}_{arriva{l}_{jkm}}\right)\times {b}_{equ{i}_k}\times {\lambda}_{VH}\times \left[1-{\left[1-{\lambda}_{VH}\times P\left(sur{v}_{km}\right)\times {b}_{equ{i}_k}\times {\lambda}_{HV}\right]}^{cul{i}_{km}}\right] $$

where $P\left(sur{v}_{arriva{l}_{jkm}}\right)$ is the probability of an infected vector surviving to its first infectious blood meal following its arrival in area *k*.

The overall national and annual median probabilities were calculated using the same procedures used to calculate the PW-host probabilities.

For a more detailed description of the probabilities and parameters used, see Additional files 2 and 3.

#### Input data

Because accurate registration data for horses were lacking, the ratio of bovines to equines per area *k* (*ρ*_*k*_ ) was estimated by combining information from different databases. The 2010 census conducted by the French Ministry of Agriculture [39] was the source for cattle and equine abundances (horses kept in agricultural settings) for each area and the IFCE-SIRE database [40] provided additional estimates of equine abundance in each area. Because it became mandatory to identify all equines in France in 2012, this database is considered to include all of the country’s equines; however, dead horses are still present in the database and, as a result, the number of equines is overestimated. Two ratios were calculated—one using each of the values of equine abundance—and *ρ*_*k*_ was estimated in our model as a uniform distribution that ranged from the smallest to the largest ratio calculated.

The number of bovines and equines transported to France were obtained from TRACES, the **TRA**de **C**ontrol and **E**xpert **S**ystem, which monitors the transport of animals and products of animal origin both into and within the EU [41]. In our analysis, we only included animals whose final destination was France.

Vector abundance was estimated using data from the national surveillance system implemented in France from 2009 to 2012—approximately 160 locations were surveyed to follow the activity of *Culicoides* populations [42]. The number of competent vectors feeding on a given equine in area *k* during month *m* (*C*_*km*_) was modeled using a truncated normal distribution; *μ* was the average monthly number of *Culicoides* collected per overnight trap (*Culicoides imicola* and members of the Obsoletus complex), *σ* was the standard deviation, and the minimum and maximum values observed were the lower and upper bounds of the distribution, respectively. Similar parameters were used in modeling efforts by de Koeijer et al. [43]. The average monthly temperature during month *m* in area *k* (*T*_*km*_) was modeled using a truncated normal distribution; *μ* was the average temperature of each month for each year (based on daily average temperatures obtained from MARS-Agri4cast)*,* and *σ* was the standard deviation*,* and the 1st and 99th percentile values were the lower and upper bounds of the distribution, respectively.

Analyses were performed for the three consecutive years included in the study: 2010, 2011, and 2012.

#### Calculations

Model calculations were performed in Microsoft Office Excel 2010 and @Risk 6.1 [44]; 10,000 iterations were run. The sensitivity analysis tool in @Risk was used to evaluate the impact of stochasticity and uncertainty in the input parameters on model results. The correlation between the values of the input parameters and the pathway-specific probabilities of introduction were calculated (Spearman’s rank correlation coefficients).

The sensitivity of the model to the values of the input parameters should have been very similar across all regions and months because we used the same model and input parameter estimates, except in the case of the bovine-to-equine ratio, the temperature data, and vector abundance. Indeed, the values of these three parameters varied across regions and months (i.e., in a given month, the vector abundance could vary greatly in one region and little in another). Larger amounts of variation could have a greater impact on the model than lesser amounts of variation. The reasoning is the same for the bovine-to-equine ratio, which also varied across regions. When determining the overall probability of introduction, we thus chose to focus our sensitivity analysis on the region-time period combinations associated with the highest levels of risk and/or uncertainty.

## Results

### Data on equine and bovine imports

TRACES data for 2010-2012 show that, on average, 1,300 equines arrived every year in France from non-EU countries, including about forty donkeys (and no zebras). Most of these animals (close to 80 %) passed through another EU country before arriving in France. Imports from high-risk regions represented an average of 1.6 % of the total imports; imports from low- and very-low-risk regions occurred at similar levels: 45.6 % and 52.4 %, respectively. By law, bovines cannot be imported from non-EU countries.

The trading of registered horses within the EU is not required to be reported to TRACES [36]. However, it is nonetheless regularly disclosed: in the TRACES database, more than 40 % of the equines traveling from other EU countries to France were registered horses. It is important to note that, in most of the data on the equine trade within the EU, no distinction is made between horses and donkeys. As a consequence, the TRACES database is somewhat limited in its ability to reveal equine movements within the EU. These concerns aside, according to the database, an average of 9,350 equines arrived in France every year from 2010 to 2012; 65 % came from very-low-risk regions, and 35 % came from low-risk regions. In the case of bovines, all movements are registered in the TRACES database. An average of 145,500 bovines arrived in France every year from 2010 to 2012; 61 % came from very-low-risk regions, and 39 % came from low-risk regions.

### Probability of release

The probability of release is defined as the probability of an infectious equine or vector being released in a given area. The overall annual median probability of release in France was 3x10^−3^ for an infectious host (PW-host) and ranged from 1.4 x10^−2^ to 3.6x10^−2^ for an infectious vector (PW-vector). Seasonal variation mostly resulted from the fact that the risk of release is negligible during the first half of the year, when low- and very-low-risk regions are considered to be unlikely to experience AHS outbreaks and equine imports from high-risk regions are very rare. From July to December across all years, the probability of release remained relatively constant; the monthly median probability that an infectious host would be released (PW-host) varied from 2.6x10^−4^ to 9.5x10^−4^, and the monthly median probability that an infectious vector (PW-vector) would be released ranged from 1.1x10^−3^ to 6.9x10^−3^. An exceptionally high peak was observed in July 2011 due to arrival of several horses from a high-risk country.

**Fig. 3:**
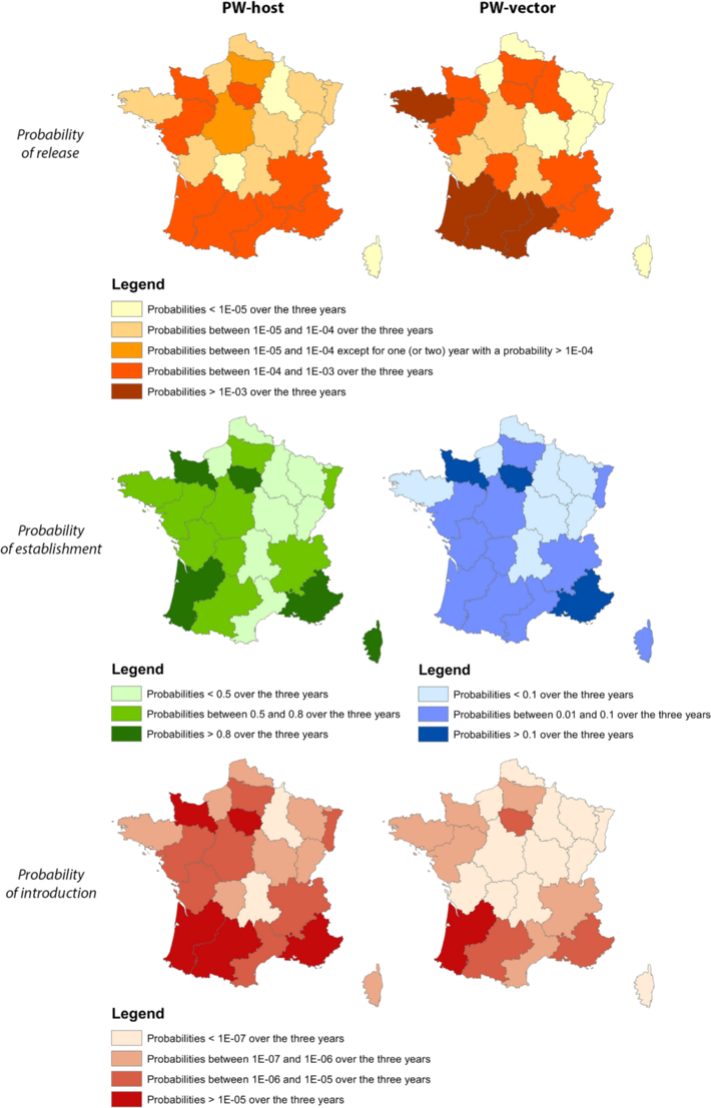
Main at risk French areas for AHSV release, establishment and introduction per introduction pathways

Areas varied greatly in their median release probabilities due to differences in the type and number of imports, but the annual probability of release for a given area was similar over time. As a result, for a given pathway of AHSV introduction, the areas most at risk remained the same (see Fig. 3).

### Probability of establishment

**Fig. 4:**
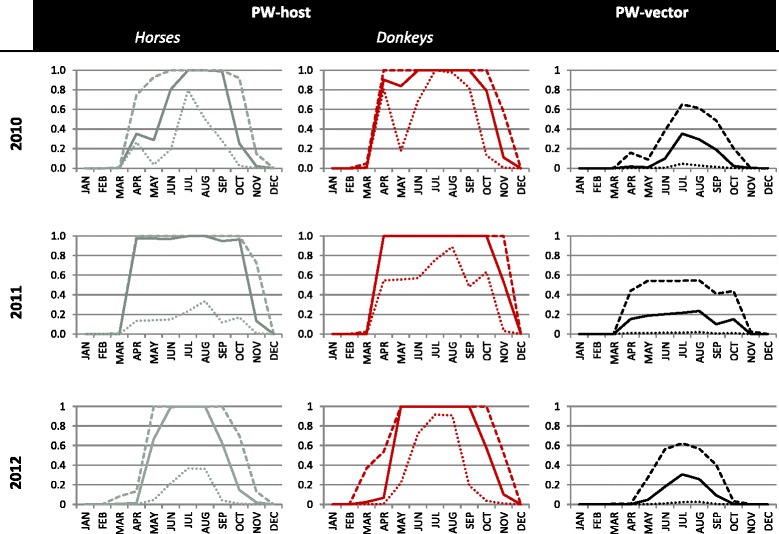
National probabilities of AHS establishment per introduction pathway and per year for France. Solid lines are the median values, large dash lines the upper border and tiny dash lines the lowest border. NB: the results for zebras are similar to results for donkeys

We determined the probability of establishment for each area of France, which was the probability that at least one local host would be infected by a local vector after the release of a single infectious host or vector. The probability of establishment varied as a function of temperature, vector abundance, the length of the equine host’s viremic period, and the bovine-to-equine ratio in arrival area *k*. In the case of the infectious vector, $P\left({estV}_{ijkm}\right)$ also depended on the life span of the specific *Culicoides* being transported. The risk of establishment was highest from May to October and peaked between June and August (Fig. 4). Temporal and regional differences were observed—owing to variation in temperature and relative host abundance—but some areas clearly faced greater risks than others (Fig. 3).

### Overall risk assessment

**Fig. 5:**
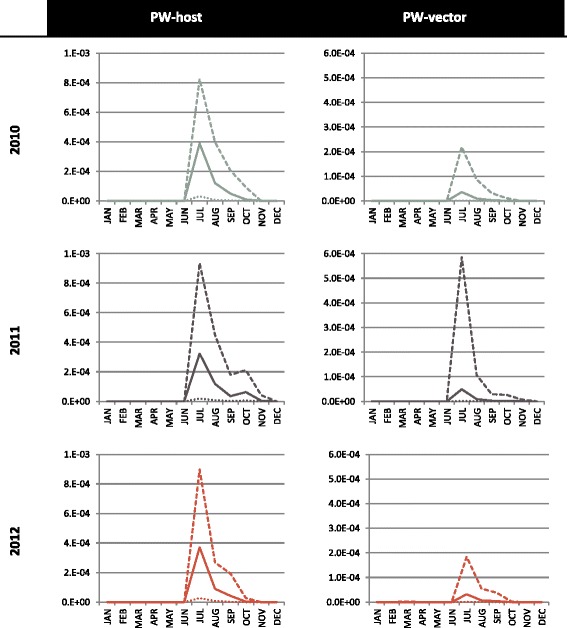
National probabilities of AHS introduction per year and per introduction pathway for France. Solid lines are the median values, large dash lines the upper border and tiny dash lines the lowest border

**Fig. 6:**
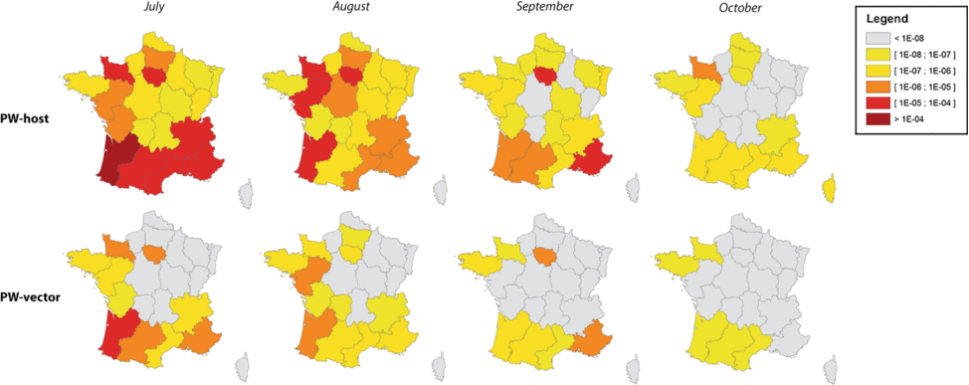
Monthly regional probabilities of AHS introduction into France from July to October 2012. Results for PW-host are presented in the upper line and results for PW-vector in the lower line. Red: probability of introduction > 1E-05; Dark orange: probability of introduction >1E-06 and < 1E-05; Light orange: probability of introduction >1E-07 and < 1E-06; Yellow: probability of introduction >1E-08 and < 1E-07; White: probability of introduction <1E-08

**Fig. 7:**
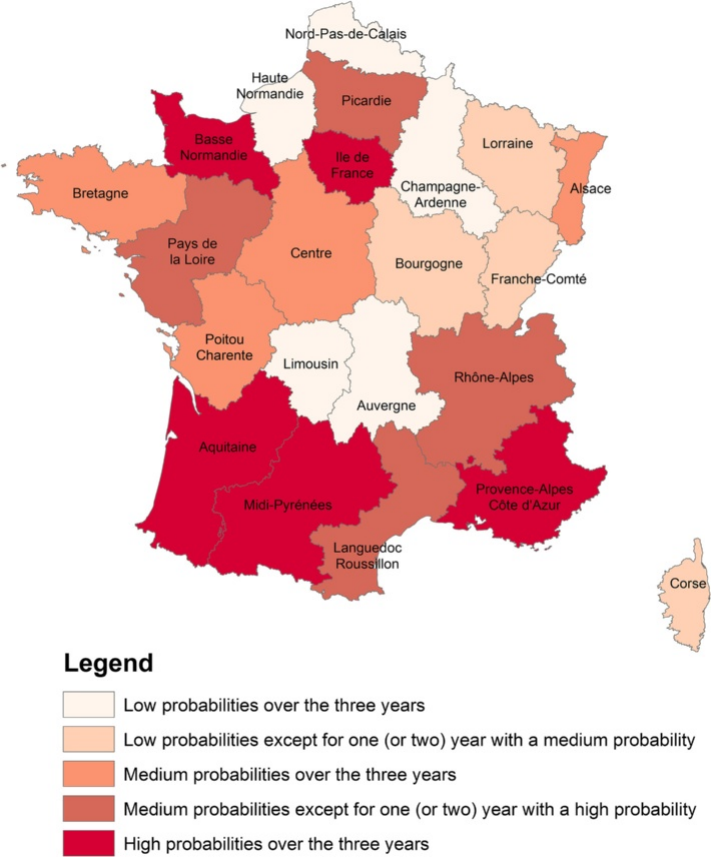
Annual regional probabilities of AHSV introduction to France via PW-host and PW-vector. Low probabilities are defined as the probabilities < 1E-06, medium probabilities as the probabilities between 1E-06 and 1E-05, high probabilities as the probabilities > 1E-05

The probability of introduction was obtained by combining the probability of release and the probability of establishment. The median annual risk of introduction due to an infectious host was almost constant across time (approximately 5x10^−4^). The median annual risk of introduction due to an infectious vector varied from 4x10^−5^ to 6x10^−5^. These figures mean that, currently, the annual risk that an infectious host will introduce AHSV into France is approximately 1 in 2,000; there is a 1 in 16,666 to 25,000 chance that AHSV introduction will be caused by an infectious vector. At the national scale, the monthly probability of introduction was similar over time, but the level of uncertainty was large (Fig. 5). The probability of introduction was the highest in the summer; it peaked in July for both pathways of introduction and in all three years. From November to June, the probability of introduction was nil, except when animals were imported from high-risk regions to the warmest areas of France (e.g., Languedoc Roussillon in March 2012). When animals were imported to colder areas, the probability of establishment was zero, making the probability of introduction zero (e.g., Basse Normandie in March 2012). Introduction risk varied greatly across space and time (see, for example, year 2012 in Fig. 6 and Additional files 4 and 5) but, over the three years examined, some areas consistently had a higher probability of introduction (see Fig. 3). If it is assumed that an average of one midge accompanies each large animal being transported, both pathways can be combined to yield a single probability of introduction (Fig. 7), to which infectious hosts appear to be the main contributors.

The average contribution of each region of origin to introduction risk is shown in Table 1. Intra-EU trade contributes most to the risk of AHSV introduction via the infectious host pathway; low-risk EU countries are the largest contributors even though they are responsible for a lower volume of imports compared with very-low-risk EU countries. This pattern is explained by the high number of equines traded within the EU and by the fact that regulations governing intra-EU trade are less strict. No zebras were brought into France during the period we studied, and donkeys represented only 0.3 % of recorded equine imports. Their average relative contribution to AHSV introduction risk was 1.2 %. Animals imported from high-risk regions account for only 0.02 % of the large livestock arriving in France, and their average contribution to AHSV introduction risk via infectious vectors was 1.5 %. As a result, imports from low-risk regions made the largest contribution to AHSV introduction risk via infectious vectors. Furthermore, cattle cannot be imported from outside the EU and, consequently, most of the transport of large livestock takes place within the EU (99.3 %). Given this fact and the fact that regulations regarding vector control are identical in all low-risk regions (EU and non-EU countries alike), trade of large livestock within the EU is the main contributor when it comes to the risk of AHSV being introduced by an infectious vector.

**Table 1:** Average contribution (%) of departure regions to the AHSV introduction risk for France. Results are presented for both pathways and compared to the total number of imports to France which are, for PW-host, the equine imports and, for PW-vector, the large animals imports (equine and bovine)

*Exporting region*		High risk	Low risk		Very low risk		TOTAL	
			*non-EU member*	*EU member*	*non-EU member*	*EU member*	*non-EU member*	*EU member*
PW-host	Risk	0.82	3.3	63.2	4.3	28	7.6	91.2
	Import	0.13	3.75	32.6	5.1	58.4	8.81	91
PW-vector	Risk	1.5	98.5		/		/	/
	Import	0.02	99.98		/		/	/

### Sensitivity analysis

**Fig. 8:**
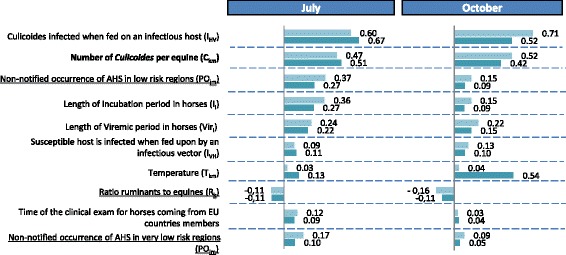
Correlation of the model input parameters with the probability of introduction of AHSV via PW-host. Results are presented for Ile de France (light blue) and Provence (dark blue) in July and October 2012. Only input parameters with at least one correlation ≥ |0.1| have been included in the tornado charts. The underline parameters are the uncertain parameters; the bold parameters are both uncertain and variable due to stochasticity; the others are only variable due to stochasticity

**Fig. 9:**
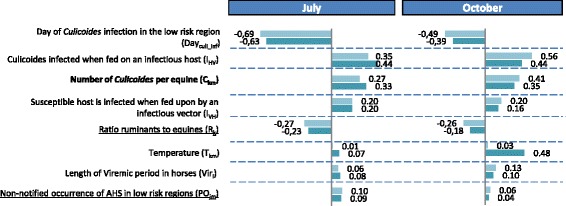
Correlation of the model input parameters with the probability of introduction of AHSV via PW-vector. Results are presented for Ile de France (light blue) and Provence (dark blue) in July and October 2012. Only input parameters with at least one correlation ≥ |0.1| have been included in the tornado charts. The underline parameters are the uncertain parameters; the bold parameters are both uncertain and variable due to stochasticity; the others are only variable due to stochasticity

A sensitivity analysis was performed for the two areas identified in Fig. 7 as being at risk for AHSV introduction via the two introduction pathways: Ile de France and Provence. The level of uncertainty surrounding the risk of introduction was rather constant for both areas, with one exception: the level of uncertainty was far higher than average in Provence in October 2012 as a result of major variation in local temperatures. The results of the analysis are summarized in Figs. 8 and 9, respectively, for the infectious host pathway and the infectious vector pathway for July (higher risk month) and October (late summer; characterized by lower risk and large uncertainty for one area) in 2012.

As expected, the values of the input parameters had a constant impact on the model’s results over time and space, with only few exceptions. Furthermore, the most important input parameters mainly encompassed variability due to stochasticity (7 out of 10 parameters for PW-host and 6 out of 8 for PW-vector) and, to a lesser extent, uncertainty. Nevertheless, compared to its effects in other areas and months, average monthly temperature had a greater impact on the results for Provence in October for both pathways of introduction. This pattern was due to the fact that temperatures varied greatly in this area during this month. This result explains the high level of uncertainty associated with the probability of introduction in this area in this month and highlights the large influence of temperature on the model’s results.

## Discussion

The model revealed that the annual risk of AHSV being introduced to France was very low and relatively constant for the pathways and years examined. The median value for the introduction risk via imported infectious equines (PW-host) was 0.0005; for infectious vectors (PW-vector), median introduction risk varied from 4x10^−5^ to 6x10^−5^ across years, assuming that one *Culicoides* midge arrived with each imported animal. The PW-host estimate was very similar to that obtained by de Vos and colleagues when they assessed the risk of AHSV being introduced to the Netherlands [24]. The latter study did not distinguish between EU countries and non-EU countries and only took into account competition horses; however, it did include horses traveling through the Netherlands to reach other countries. The PW-vector estimate was highly dependent on our assumption that only one *Culicoides* midge was associated with each imported animal. Indeed, the higher the number of associated *Culicoides*, the higher the probability of introduction. If it is the case that, on average, one *Culicoides* is associated with each imported animal, then the risk tied to this pathway of introduction is ten times lower than that tied to infectious hosts. As a result, AHS-free countries only face significant introduction risks if the number of *Culicoides* being transported is large as that required for BTV, as shown by Napp et al. for Spain [19]. On the one hand, our assumption of one *Culicoides* per animal could be overly pessimistic because midges could exit the transport vehicle after feeding and thus not reach the animal’s final destination. On the other hand, it could be an overly optimistic assumption because large numbers of *Culicoides* may be found on large animals. Because data are lacking on the number of *Culicoides* being transported with mammalian hosts, it is difficult to determine how each pathway of introduction contributes to overall introduction risk.

Our study indicates AHSV establishment in France may be favored from May to October. This finding is consistent with the results obtained by Lo Iacono et al. for the UK [45], by Martinez-Lopez et al. for Spain [46] and by de Vos et al. for the Netherlands [24]. In France, the favorable period for AHSV establishment is longer than in the UK (June to September) and the Netherlands (June to August) but shorter than in Spain (April to December), which is a logical consequence of climatic differences. Such differences should be taken into account when AHSV introduction within Europe is addressed at a larger scale.

Major differences were found among French regions and between introduction pathways. As expected, the coldest regions with the smallest equine populations had the lowest risk of AHSV introduction (e.g., Centre and Auvergne). In contrast, the warmest regions were most at risk for AHSV introduction (e.g., Aquitaine and Midi-Pyrénées), as were colder regions with larger equine populations (e.g., Basse Normandie and Ile de France). Warmer regions faced higher levels of risk mostly because of their favorable climatic conditions, while larger equine populations increased risk in colder regions. Nevertheless, if spatial differences were mainly determined by the probability of establishment, import-related variation (number and species imported) also played an important role, as seen in the *Corse* region: even if climatic conditions are favorable, the probability of introduction will be low if there are very few imported animals. These results emphasize that it is important to analyze spatiotemporal variation in risk when developing efficient surveillance systems and optimizing control measures. For instance, if there is a high risk that a pathogen will be introduced by an infectious vector, insecticides should be applied before animals are imported. In contrast, if there is a high risk that a pathogen will be introduced by an infectious host, quarantine measures should be more stringent and/or extra tests should be performed on horses coming from low-risk regions.

We found that seasonal variation in temperature can have a large impact on the risk of introduction because it exerts a strong influence over vector abundance and biology, which, taken together, determine a vector’s capacity to transmit AHSV [3, 47]. The risk of ASHV introduction was higher during periods characterized by higher than average temperatures. This finding concurs with results from work examining the introduction of BTV-8 to northwestern Europe: the extreme temperatures during July 2006 may have contributed to its widespread diffusion [48]. Therefore, rare, extreme climatic events and, more generally, global warming should have a large influence on the probability of AHSV establishment, as has been shown for BTV [49, 50]. Given the progression of global warming, risk assessments should be regularly updated to account for climatic changes.

Our study reveals that complete and accurate data on the movements and distribution of the EU’s equine population are not available: it is hard to trace horses within the EU. The introduction of mandatory horse passports in 2008 improved the situation but, apart from rare exceptions [46], it is still difficult to follow the EU’s equine population [51] and assessing the population’s distribution and fluxes remains a challenge (see the UK [52]). for an example). This is a major concern given that the distribution of the equine population had an important impact on our results (see the ratio of bovines to equines in Figs. 8 and 9); moreover, the number of equines being imported is obviously highly correlated with introduction probabilities. Furthermore, several equine viruses are zoonotic (e.g., eastern and western equine encephalomyelitis viruses, Venezuelan equine encephalitis virus, and West Nile virus), and the risk that they will be introduced to and spread within the EU is definitely not negligible [22]. It is, in fact, currently increasing; indeed, West Nile virus has already become endemic in some regions [53]. Improving the traceability of horses within the EU would thus be advantageous when it comes to better assessing the risk posed by AHSV and other zoonotic diseases.

The risk assessment model described in this paper addresses the risk of AHSV being introduced to France by two pathways of introduction considered to be of importance [23]. However, other pathways may also substantially contribute to introduction risk. Several studies have highlighted that the wind may efficiently transport *Culicoides* over long distances, both across sea [25, 54] and land [55]; it might have been involved in the spread of BTV in Europe [25–27]. Wind-mediated dispersal of infected vectors might also have resulted in AHSV being introduced to Cape Verde Island in 1944, Cyprus in 1960, the Middle East in 1960 and Spain in 1966 [56]. An extensive assessment of the role played by the wind in spreading *Culicoides* midges and *Culicoides*-borne infections across the Mediterranean Basin would elucidate the importance of this pathway for AHS introduction. However, the wind-mediated dispersal of AHSV is most likely to occur in the south of France close to low-risk regions, which is also where AHSV introduction is most likely to occur via the pathways examined in this study. Therefore, we think that this study accurately identifies the regions of France that face the greatest risk of AHSV introduction. Furthermore, the risk that AHSV will be introduced via wind-borne infectious *Culicoides* cannot be mitigated by direct preventive measures, such as importation restrictions. Instead, to be effective, control measures would have to influence the probability of establishment; for instance, insecticides could be used to protect local hosts against wind-borne vectors.

Our model provides a basis for creating a risk-based surveillance system in France that focuses on the regions and time periods associated with higher levels of AHSV introduction risk. The model could also be used for assessing risk and establishing surveillance procedures in other European countries. This application is especially important because our study has revealed that European countries make the largest contribution to France’s AHSV introduction risk (e.g., PW-host: 91.2 %). Indeed, if an infection occurs in one European country but is not detected, then it can easily spread to other European countries because there is little verification and tracking of equine movements within the EU. By implementing a risk-based surveillance strategy in each country in the EU, infections would have a higher probability of being detected early on; as a consequence, the contribution of fellow EU countries to introduction risk would decline (see the probabilities of non-notified AHS occurrence in low- and very-low-risk regions in Figs. 8 and 9). By reinforcing the tracking of equine movements within the EU, infection would also have less chance to disseminate and the policy implications of an AHS introduction will be more limited. Such strategies could be a means of minimizing the risk and impact of an AHSV outbreak for the entire European equine industry.

## Conclusion

We have developed a quantitative risk assessment model to estimate the risk of AHSV being introduced to France via the importation of infectious equines and infectious *Culicoides* midges associated with large livestock. The risk that AHS will be introduced to France is very low; however, risk varies tremendously among the different regions of the country due to variation in temperature and equine population size. The regions most at risk are those with the warmest climates as well as those that are colder but that harbor larger equine populations. Introduction risk is greatest from July to October and peaks in July. Despite the low probability that AHSV is present in the EU, intra-EU trade of equines contributes most to the risk of AHSV introduction to France because it is responsible for a large number of horse movements. Spatiotemporal differences need to be addressed when assessing the risk that AHSV will be introduced to a given location and when developing and implementing risk-based surveillance procedures. The methods and results of this study may help guide surveillance programs and other risk-reduction measures aimed at preventing the introduction of AHSV or minimizing its potential impact once it has been introduced, both in France and in other European countries.

## Additional files

Additional file 1:
**Model calculation for PW-host.** Details of calculation regarding the AHSV introduction via the import of an infectious host. Additional file 2:
**Model parameters.** Description of all parameters used in the model calculation for PW-host and PW-vector. Additional file 3:
**Model calculation for PW-vector.** Details of calculation regarding the AHSV introduction via the import of an infectious vector. Additional file 4:
**Spatiotemporal risk of AHSV introduction via PW-host to France from 2010 to 2012.**
Additional file 5:
**Spatiotemporal risk of AHSV introduction via PW-vector to France from 2010 to 2012.**
