# Supplementary material for: A spatiotemporal model to assess the introduction risk of African horse sickness by import of animals and vectors in France
Source: BMC Vet Res. 2015 Jun 4;11:127. doi: 10.1186/s12917-015-0435-4 (PMC4455332; doi:10.1186/s12917-015-0435-4)
Supplement: Additional file 1: — Model calculation for PW-host. Details of calculation regarding the AHSV introduction via the import of an infectious host. [file 12917_2015_435_MOESM1_ESM.docx]

**Additional file 1: Model calculation for PW-host**

Every calculus below is made for an equine from species *i* imported from an area *j* to the free area *k* the month *m*. All the parameters used are detailed in the Additional file 2.

The probability of introduction for PW-host is the probability to import at least one infected host able to transmit the infection to at least one local host and is defined as:

$$P\left( \mathrm{introH}_{\mathrm{ijkm}} \right)=1-\left[ 1-P\left( \mathrm{relH}_{\mathrm{ijkm}} \right)\times P\left( \mathrm{estH}_{\mathrm{ijkm}} \right) \right]^{\mathrm{eq}_{\mathrm{ijkm}}}$$

Where $P\left( \mathrm{relH}_{\mathrm{ijkm}} \right)$, the probability of release, depends of the importation procedure implemented and the periods where a host is infected and$P\left( \mathrm{estH}_{\mathrm{ijkm}} \right)$, the probability of establishment, is defined as:

$P\left( \mathrm{estH}_{\mathrm{ijkm}} \right)=1-{[1-I_{VH}\times P(surv_{km})\times b_{equi_{k}}\times I_{HV}]}^{{culi}_{km}}$

with *culi_km_* the number of vectors feeding on an infected viraemic imported host equals at BR_km_ x Vir x C_km_

For each category of exporting region, there is different import procedure implemented and thus different periods *z* where a host can be infected. For a given region *j*, there is a total of *w* different time periods *z* where the equine can be infected depending on the import procedure implemented for the region *j.* The different periods *z* for each region *j* are presented below:

- **High risk countries:** host can be infected 1) Before quarantine, 2) During quarantine but before the first serological test CF1, 3) During quarantine but between the both serological tests CF1 and CF2, 4) During quarantine but after CF2 and before clinical exam, or 5) After clinical exam.
- **Low risk countries:**
  - **Non EU country member:** host can be infected 1) Before quarantine, 2) During quarantine but before CF1, 3) During quarantine but between CF1 and CF2, 4) During quarantine but after CF2 and before clinical exam, or 5) After clinical exam.
  - **EU country member:** host can be infected 1) Before clinical exam, or 2) After clinical exam.
- **Very low risk countries:**
  - **Non EU country member:** host can be infected 1) Before clinical exam, or 2) After clinical exam.
  - **EU country member:** host can be infected 1) Before clinical exam, or 2) After clinical exam.

The probability of release by species *i* from region *j* to area *k* during a specific month *m* ($P\left( \mathrm{relH}_{\mathrm{ijkm}} \right))$ is thus calculated as: $P\left( \mathrm{relA}_{\mathrm{ijkm}} \right)=\frac{\sum_{z=1}^{w} [\left( length period z \right)\times P\left( \mathrm{relA}_{\mathrm{ijkmz}} \right)]}{\sum_{z=1}^{w} \left( length period z \right)}$

Where $P\left( {relH}_{ijkmz} \right)$is the probability of release when the animal *i* is infected during the time period *z*.

P(relA_ijkmz_) is calculated for each period *z* as:

$$P\left( \mathrm{relA}_{\mathrm{ijkmz}} \right)=P(\inf_{\mathrm{ijmz}})\times P(\mathrm{vir}_{\mathrm{ijmz}})\times(1-{P(CF1}_{\mathrm{iz}}))\times(1-{P(CF2}_{\mathrm{iz}}))\times(1-{P(clin}_{\mathrm{ijmz}}))\times(1-{P(trans}_{\mathrm{ijkz}}))$$

1. $\mathbf{P}\left( \mathbf{inf}_{\mathbf{ijmz}} \right)\boldsymbol{=}$**Probability for a host to be infected during period z in the month *m* in area *j***

The probability of infection during a certain period *z* (before or during the import procedure) depends on the fraction of this period *z* spend in each of the months *m*, *m-1* and *m-2*.

1. No quarantine and CF test are required

Entire period of being at risk of infection is the high risk period (HRP).

- ***Probability that the imported host is infected before clin***

If HRP < e

$$=\mathrm{PO}_{\mathrm{jm}}\times CI_{m}$$

If HRP > e

If HRP < 30 + e

$=\frac{\mathrm{PO}_{\mathrm{jm}}}{\mathrm{HRP}} \times[\mathrm{CI}_{m} \times e + \mathrm{CI}_{m-1}\times(HRP - e)]$

If HRP > 30 + e

$=\frac{\mathrm{PO}_{\mathrm{jm}}}{\mathrm{HRP}} \times[\mathrm{CI}_{m}\times e + \mathrm{CI}_{m-1}\times30 + \mathrm{CI}_{m-2}\times(HRP-30-e)]$

- ***Probability that the imported host is infected after clin***

$$=\mathrm{PO}_{\mathrm{jm}}\times CI_{m}$$

1. Quarantine and CF tests required

- ***Probability that the imported host is infected before q***

If q – e < 30

$$=\frac{\mathrm{PO}_{\mathrm{jm}}}{\mathrm{Inf}_{\mathrm{time}}} \times[\mathrm{CI}_{m-1}\times(30-q+e)+\mathrm{CI}_{m-2}\times(\mathrm{Inf}_{\mathrm{time}}-(30-q+e))]$$

If q – e > 30

$$=\frac{\mathrm{PO}_{\mathrm{jm}}}{\mathrm{Inf}_{\mathrm{time}}} \times[\mathrm{CI}_{m-2}\times(60-q+e)+\mathrm{CI}_{m-3}\times(\mathrm{Inf}_{\mathrm{time}}-60+q-e)]$$

- ***Probability that the imported host is infected between q and cf1***

If e – cf1 < 0

If q > 30 + e

$$=\frac{\left( 1-Prot_{\mathrm{vect}} \right)\times\mathrm{PO}_{\mathrm{jm}}}{q-cf1} \times[\mathrm{CI}_{m-1}\times(30-cf1+e)+\mathrm{CI}_{m-2}\times(q-30-e)]$$

If q < 30 + e

$$=\left( 1-\mathrm{Prot}_{\mathrm{vect}} \right)\times\mathrm{PO}_{\mathrm{jm}}\times\mathrm{CI}_{m-1}$$

if e – cf1 > 0

if q > 30 + e

$=\frac{\left( 1-Prot_{\mathrm{vect}} \right) \times\mathrm{PO}_{\mathrm{jm}}}{q-cf1} \times[\mathrm{CI}_{m}\times(e-cf1)+\mathrm{CI}_{m-1}\times30+\mathrm{CI}_{m-2}\times(q-30-e)]$

if q < 30 + e

$$=\frac{\left( 1-Prot_{\mathrm{vect}} \right) \times\mathrm{PO}_{\mathrm{jm}}}{q-cf1} \times[\mathrm{CI}_{m}\times(e-cf1)+\mathrm{CI}_{m-1}\times(q-e)]$$

- ***Probability that the imported host is infected between cf1 and cf2***

If e < cf2

$$=\frac{\left( 1-Prot_{\mathrm{vect}} \right) \times\mathrm{PO}_{\mathrm{jm}}}{cf1-cf2} \times[\mathrm{CI}_{m-1}\times(30-cf2+e)+\mathrm{CI}_{m-2}\times(cf1-30-e)]$$

If e > cf2

if cf1 > 30 + e

$$=\frac{\left( 1-Prot_{\mathrm{vect}} \right) \times\mathrm{PO}_{\mathrm{jm}}}{cf1-cf2} \times[\mathrm{CI}_{m}\times(e-cf2)+\mathrm{CI}_{m-1}\times30+\mathrm{CI}_{m-2}\times(cf1-30-e)]$$

if cf1 < 30 + e

$$=\frac{\left( 1-Prot_{\mathrm{vect}} \right) \times\mathrm{PO}_{\mathrm{jm}}}{cf1-cf2} \times[\mathrm{CI}_{m}\times(e-cf2)+\mathrm{CI}_{m-1}\times(cf1-e)]$$

- ***Probability that the imported host is infected after cf2***

If e < cf2 $=\frac{\left( 1-Prot_{\mathrm{vect}} \right) \times\mathrm{PO}_{\mathrm{jm}}}{cf2} \times[\mathrm{CI}_{m}\times e+\mathrm{CI}_{m-1}\times(cf2-e)]$

If e > cf2

$=(1-\mathrm{Prot}_{\mathrm{vect}} ) \times\mathrm{PO}_{\mathrm{jm}}\times\mathrm{CI}_{m}$

1. $\mathbf{P}\left( \mathbf{vir}_{\mathbf{ijmz}} \right)\mathbf{=}$**Probability for a host to be vireamic or incubating when imported to area B given being infected**

Calculation is based on a constant viraemic and latent period, which is equal for each equine of species *i*.

1. No quarantine and CF test are required

- ***When infected before clin***

If In + Vir < t_AB_ + clin

= 0

If In + Vir > HRP + t_AB_ + clin

= 1

If In + Vir < HRP + t_AB_ + clin

$$=\frac{In+Vir-t_{\mathrm{AB}}}{HRP-clin}$$

- ***When infected after clin***

If In > t_AB_ + clin

= 1

If In < t_AB_ + clin

If In + Vir > t_AB_ + clin

= 1

If In + Vir < t_AB_ + clin

$$=\frac{In+Vir}{t_{\mathrm{AB}}+clin}$$

1. Quarantine and CF tests required

- ***When infected before q***

If In + Vir > Inf_time_ + q + t_AB_

= 1

If In + Vir < q + t_AB_

= 0

If In + Vir < Inf_time_ + q + t_AB_

$$=\frac{In+Vir-q-t_{\mathrm{AB}}}{\mathrm{Inf}_{\mathrm{time}}}$$

- ***When infected between q and cf1***

If In + Vir > q + t_AB_

= 1

If In + Vir < cf1 + t_AB_

= 0

If q + t_AB_ > In + Vir > cf1 + t_AB_

$$=\frac{In+Vir-cf1-t_{\mathrm{AB}}}{q-cf1}$$

- ***When infected between cf1 and cf2***

If In + Vir > cf1 + t_AB_

= 1

If In + Vir < cf2 + t_AB_

= 0

If cf1 + t_AB_ > In + Vir > cf2 + t_AB_

$$=\frac{In+Vir-cf2-t_{\mathrm{AB}}}{cf1-cf2}$$

- ***When infected after cf2***

If In + Vir > cf2 + t_AB_

= 1

If In + Vir < t_AB_

= 0

If cf2 + t_AB_ > In + Vir > t_AB_

$$=\frac{In+Vir-t_{\mathrm{AB}}}{cf2}$$

1. $\mathbf{P(clin}_{\mathbf{ijmz}}\mathbf{)=}$**Probability for an infected host to be detected during importation procedure**
2. No quarantine and CF test are required

Probability to be detected during importation procedure = Probability to be detected by clinical inspection

- ***When infected before clin***

If In > HRP – clin

= 0

If In < HRP – clin

If In + Vir < HRP – clin

$$=\frac{Vir\times\mathrm{Se}_{\mathrm{clin}}}{HRP-clin}$$

If In + Vir > HRP – clin

$$=\frac{(HRP-clin-In)\times\mathrm{Se}_{\mathrm{clin}}}{HRP-clin}$$

- ***When infected after clin***

= 0

1. Quarantine and CF tests required
2. ${P(CF1}_{iz})=$*Probability for an infected host to be detected by cf1*

- ***When infected before q***

if q – cf1 < Sero

= Se

If Inf_time_ + q – cf1 < Sero

= 1 – Sp

If Inf_time_ + q – cf1 > Sero

$$=\frac{(\mathrm{Inf}_{\mathrm{time}}-Sero+q-cf1)\times Se}{\mathrm{Inf}_{\mathrm{time}}}+\frac{(Sero-q+cf1)\times(1-Sp)}{\mathrm{Inf}_{\mathrm{time}}}$$

- ***When infected between q and cf1***

If q – cf1 < Sero

= 1 – Sp

if q – cf1 > Sero

$$=\frac{(q-cf1-Sero)\times Se}{q-cf1}+\frac{Sero\times(1-Sp)}{q-cf1}$$

1. ${P(CF2}_{iz})=$*Probability for an infected host to be detected by cf2*

*Assumption: cf1 and cf2 are independent*

- ***When infected before q***

if q – cf2 > Sero

= Se

If Inf_time_ + q – cf2 < Sero

= 1 – Sp

If Inf_time_ + q – cf2 > Sero

$$=\frac{(\mathrm{Inf}_{\mathrm{time}}-Sero+q-cf2)\times Se}{\mathrm{Inf}_{\mathrm{time}}}+\frac{(Sero-q+cf2)\times(1-Sp)}{\mathrm{Inf}_{\mathrm{time}}}$$

- ***When infected between q and cf1***

if q – cf2 < Sero

= 1 – Sp

If q – cf2 > Sero

If cf1 – cf2 > Sero

= Se

If cf1 – cf2 < Sero

$$=\frac{(q-cf2-Sero)\times Se}{q-cf1}+\frac{(Sero-cf1+cf2)\times(1-Sp)}{q-cf1}$$

- ***When infected between cf1 and cf2***

if cf1 – cf2 < Sero

= 1 – Sp

If cf1 – cf2 > Sero

$$=\frac{(cf1-cf2-Sero)\times Se}{cf1-cf2}+\frac{Sero\times(1-Sp)}{cf1-cf2}$$

1. *P(clin) = Probability for an infected host to be detected by clinical inspection*

- ***When infected before q***

If In + Vir < q – clin or In > Inf_time + q – clin

= 0

If In + Vir > Inf_time + q – clin

If In < q – clin

= Se_clin_

If In > q – clin

$$=\frac{(In-q+clin)\times\mathrm{Se}_{\mathrm{clin}}}{\mathrm{Inf}_{\mathrm{time}}}$$

If Inf_time + q – clin > In + Vir > q – clin

If In < q – clin

$$=\frac{(In+Vir-q+clin)\times\mathrm{Se}_{\mathrm{clin}}}{\mathrm{Inf}_{\mathrm{time}}}$$

If In > q – clin

$$=\frac{Vir\times\mathrm{Se}_{\mathrm{clin}}}{\mathrm{Inf}_{\mathrm{time}}}$$

- ***When infected between q and cf1***

If In + Vir < cf1 – clin or In > q – clin

= 0

If In + Vir > q – clin

If In < cf1 – clin

= Se_clin_

If In > cf1 – clin

$$=\frac{(In-cf1+clin)\times\mathrm{Se}_{\mathrm{clin}}}{q-cf1}$$

If q – clin > In + Vir > cf1 – clin

If In < cf1 – clin

$$=\frac{(In+Vir-cf1+clin)\times\mathrm{Se}_{\mathrm{clin}}}{q-cf1}$$

If In > cf1 – clin

$$=\frac{Vir\times\mathrm{Se}_{\mathrm{clin}}}{q-cf1}$$

- ***When infected between cf1 and cF2***

If In + Vir < cf2 – clin or In > cf1 – clin

= 0

If In + Vir > cf1 – clin

If In < cf2 – clin

= Se_clin_

If In > cf2 – clin

$$=\frac{(In-cf2+clin)\times\mathrm{Se}_{\mathrm{clin}}}{cf1-cf2}$$

If cf1 – clin > In + Vir > cf2 – clin

If In < cf2 – clin

$$=\frac{(In+Vir-cf2+clin)\times\mathrm{Se}_{\mathrm{clin}}}{cf1-cf2}$$

If In > cf2 – clin

$=\frac{Vir \times\mathrm{Se}_{\mathrm{clin}}}{cf1-cf2}$

- ***When infected after cf2***

If In > cf2 – clin

= 0

If In < cf2 – clin

If In + Vir > cf2 – clin

$=\frac{\left( cf2- clin-In \right)\times\mathrm{Se}_{\mathrm{clin}}}{cf2-clin}$

If In + Vir < cf2 – clin

$$=\frac{Vir\times\mathrm{Se}_{\mathrm{clin}}}{cf2-clin}$$

1. $\mathbf{P(trans}_{\mathbf{ijkz}}\mathbf{)=}$**Probability for an infected host to be detected during transport from A to B given having passed the examinations and testing prior to embarkation.**
2. No quarantine and CF test

- ***When infected before clin***

If In > HRP + t_AB_ - clin

= 0

If In < HRP + T_AB_ - clin

If In + Vir < HRP – clin

$$=\frac{Vir\times\mathrm{Se}_{\mathrm{clin}}}{HRP-clin}$$

If In + Vir > HRP – clin

$$=\frac{(HRP-clin+t_{\mathrm{AB}}-In)\times\mathrm{Se}_{\mathrm{clin}}}{HRP-clin}$$

- ***When infected after clin***

If In > t_AB_ + clin

= 0

If In < t_AB_ + clin

If In + Vir < clin + t_AB_

$$=\frac{Vir\times\mathrm{Se}_{\mathrm{clin}}}{t_{\mathrm{AB}}-clin}$$

If In + Vir > clin + t_AB_

$$=\frac{(clin+t_{\mathrm{AB}}-In)\times\mathrm{Se}_{\mathrm{clin}}}{t_{\mathrm{AB}}-clin}$$

1. Quarantine and CF tests required

- ***When infected before q***

If In + Vir < q + t_AB_ or In > Inf_time + q + t_AB_

= 0

If In + Vir > Inf_time + q + t_AB_

If In > q + t_AB_

$$=\frac{(In-q-t_{\mathrm{AB}})\times\mathrm{Se}_{\mathrm{clin}}}{\mathrm{Inf}_{\mathrm{time}}}$$

If In < q + t_AB_

= Se_clin_

If Inf_time + q + t_AB_ > In + Vir > q + t_AB_

If In > q + t_AB_

$$=\frac{Vir\times\mathrm{Se}_{\mathrm{clin}}}{\mathrm{Inf}_{\mathrm{time}}}$$

If In < q + t_AB_

$$=\frac{(In-q-t_{\mathrm{AB}})\times\mathrm{Se}_{\mathrm{clin}}}{\mathrm{Inf}_{\mathrm{time}}}$$

- ***When infected between q and cf1***

If In + Vir < cf1 + t_AB_ or In > q + t_AB_

= 0

If In + Vir > q + t_AB_

If In > cf1 + t_AB_

$$=\frac{(In-cf1-t_{\mathrm{AB}})\times\mathrm{Se}_{\mathrm{clin}}}{q-cf1}$$

If In < cf1 + t_AB_

= Se_clin_

If q + t_AB_ > In + Vir > cf1 + t_AB_

If In > cf1 + t_AB_

$$=\frac{Vir\times\mathrm{Se}_{\mathrm{clin}}}{q-cf1}$$

If In < cf1 + t_AB_

$$=\frac{(In+Vir-cf1-t_{\mathrm{AB}})\times\mathrm{Se}_{\mathrm{clin}}}{q-cf1}$$

- ***When infected between cf1 and cf2***

If In + Vir < cf2 + t_AB_ or In > cf1 + t_AB_

= 0

If In + Vir > cf1 + t_AB_

If In > cf2 + t_AB_

$$=\frac{(In-cf2-t_{\mathrm{AB}})\times\mathrm{Se}_{\mathrm{clin}}}{cf1-cf2}$$

If In < cf2 + t_AB_

= Se_clin_

If cf1 + t_AB_ > In + Vir > cf2 + t_AB_

If In > cf2 + t_AB_

$$=\frac{Vir\times\mathrm{Se}_{\mathrm{clin}}}{cf1-cf2}$$

If In < cf2 + t_AB_

$$=\frac{(In+Vir-cf2-t_{\mathrm{AB}})\times\mathrm{Se}_{\mathrm{clin}}}{cf1-cf2}$$

- ***When infected after cf2***

If In > cf2 + t_AB_

= 0

If In < cf2 + t_AB_

If In + Vir > cf2 + t_AB_

$$=\frac{(cf2+t_{\mathrm{AB}}-In)\times\mathrm{Se}_{\mathrm{clin}}}{cf2}$$

If In + Vir > cf2 + t_AB_

$$=\frac{Vir\times\mathrm{Se}_{\mathrm{clin}}}{cf2}$$

1. $\mathbf{P}\left( \mathbf{sur}\mathbf{v}_{\mathbf{km}} \right)\mathbf{=}$**Probability that the vector survives to the EIP and can have a blood meal during the month *m***

$$P\left( \mathrm{sur}v_{\mathrm{km}} \right)= e^{-(N_{km}\times GC_{km}\times MR_{km})}$$
