# Supplementary material for: A spatiotemporal model to assess the introduction risk of African horse sickness by import of animals and vectors in France
Source: BMC Vet Res. 2015 Jun 4;11:127. doi: 10.1186/s12917-015-0435-4 (PMC4455332; doi:10.1186/s12917-015-0435-4)
Supplement: Additional file 2: — Model parameters. Description of all parameters used in the model calculation for PW-host and PW-vector. [file 12917_2015_435_MOESM2_ESM.docx]

**Additional file 2: Model parameters**

Preliminary definitions:

- T_km_ is the average monthly temperature during the month m in the area k (details of the estimation available in the main text)
- C_km_ is the number of competent vectors feeding on one equine in area *k* during the month *m* (details of the estimation available in the main text)
- *ρ_k_* is the ratio of the number bovines to the number of equines per area *k* (details of the estimation available in the main text)
- e is defined as the day of embarkation (set the 12^th^ of each month)
- q is the length of quarantine (40 days given EU regulation)
- clin is the day of the clinical exam before embarkation (equals zero, one or two days depending on the departure region given EU regulation).
- Cf1 is the day when the first test is performed (35 days before embarkation given EU regulation)
- Cf2 is the day when the second test is performed (10 days before embarkation given EU regulation)

| **PARAMETER** | | **ESTIMATION** | | **REFERENCES** |
| --- | --- | --- | --- | --- |
| ***Vertebrate hosts*** | | |  |  |
| *I_i_* | Incubation period (days) | All equines: Pert(2,6,10) | | [1] [2] |
| *Vir_i_* | Viraemic period (longer for surviving animals than for animals succumbing from disease) | Horses: Discrete({Gamma(29.75,0.20). Gamma(20.25,0.22)};{0.3,0.7})  Donkeys: Discrete({28,12};{0.9,0.1})  Zebras: Discrete({40,28};{0.99,0.01}) | | [1] [3] |
| *Sero_i_* | Time to seroconvertion (days) | All equines: Uniform(10,14) | | [1] |
| ***Vectors*** | | |  |  |
| *GC_km_* | Length of the gonotrophic cycle during the month *m* (days) | = -1.98 + 0.07217× T_km_ + 2516.65/T_km_^2^ | | [4] |
| *BR_km_* | Biting rate = reciprocal of the blood feeding interval (= Gonotrophic cycle) | = 0.015 × T_km_ – 0.125 | | [4] [3] |
| *EIP_km_* | Length of the EIP (days) | = 0.0085 × T_km_ – 0.0821 | | [4] |
| *MR_km_* | Mortality rate of the vector (days^-1^) | = 0.015 × exp(0.063 × T_km_) | | [1] [3] [4] |
| *N_km_* | Number of gonotrophic cycles to complete an EIP + time to next blood meal | = Roundup(EIP_km_/GC_km_) | |  |
| ***Interaction host vector*** | | |  |  |
| *λ_HV_* | Probability for a vector to become infected after feeding on a viraemic host | All equines: Beta(1.05,39.6) with a mean value of 0.02 | | [5] [6] |
| *λ_VH_* | Probability for a host to become infected after being bitten by an infectious vector | All equines: Beta(6,2) with a mean value of 0.77 | | [1] [7] [3] |
| ***Export regulations*** | | | | |
| *Se* | Sensitivity of the CF test | Beta(60,4) | | [1] |
| *Sp* | Specificity of the CF test | Beta(62,2) | | [1] |
| *Se_clin_* | Sensitivity of clinical examination | Horses 0.7; Donkeys 0.1; Zebras 0.01 | | [1] [8] |
| *Prot_vect_* | Efficiency of protection against vectors | Uniform (0.5,0.9) | | [1] |
| *D_transp_* | Day of vector transportation after infection | Uniform(*D_culi_inf_* ; 1/MR_km_) | |  |
| ***Departure region j*** | | | | |
| *PO_jm_* | Probability of disease occurrence | Endemic: 1  Low risk: Gamma [(15 x HRP_2_), 1/(60 x 365)]  Very low risk: Gamma [HPR_3_, 1/(61 x 365)] | | [1] |
| ***Arrival area k*** | | | | |
| *b_equi_* | Probability for a vector to bite a susceptible host | = 1/(a x *ρ*_k_ + 1)  With, *a* the vector preference for equidae (We assume that the vector has no host preference between cattle and horse and that *a* = 1) | | [9] [10][11] |

***Inf_time_ =* Period when a horse can be infected before the start of import procedure such as quarantine or clinical exam when there is no quarantine**

If a quarantine applied: if HRP – q > 0, Inf_time_ = HRP – q but if HRP – q < 0, Inf_time_ = 0.

If no quarantine applied: Inf_time_ = HRP – clin

**HRP = High risk period**

The HRP is the time between virus introduction and the first formal detection. In low and very low risk region, we assumed that the first infected horse won’t be detected but that the secondary cases will be. Thus the time needed to detect the second case is estimated as the time required for two incubation periods plus the time till the next infectious blood meal of a vector. In low risk regions HRP_2_ is assumed equal at 22 days (based on a temperature in the region *j* of 18°C). In very low risk region, HRP_3_ is assumed equal at 60 days (based on a temperature in the region *j* of 12°C). As in high risk region the virus is supposed endemic, there is no real HRP_1_ because an equine can be infected at any time (during or before quarantine). We thus choose to set a period of 30 days before the start of quarantine (thus 70 days before embarkation) as the earliest stage when a host can be infected.

***D_culi_inf_* = Day of vector becomes infected**

To estimate the day where the vector becomes infected (*D_culi_inf_* ), we first only take into account the *Culicoides* susceptible to the infection. When a *Culicoides* is susceptible, one blood meal on a vireamic host is assumed sufficient for this vector to become infected [12]. Assuming a uniform distribution of the viraemic host and a constant monthly temperature T_jm_ in each departure area *j*, the moment (or day) of *Culicoides* infection follows a Uniform distribution between 1 (the *Culicoides* is infected the first day of its life) and 1/MR_km_ (the *Culicoides* is infected the last day of its life).

***CI_ijm_* = Cumulative monthly number of infectious hosts *i* in each departure area *j***

Equidae have a seasonal foaling period but the foaling season depends on the geographical area considered (North or South hemisphere). We thus assumed that *CI_ijm_* was a constant for all species in all departure region *j*. For low and very low risk regions, *CI_ijm_* was considered as equals at 2x10^-4^ for all species based on AHSV epidemic in Spain [13, 14] [1]. For high risk region, *CI_ijm_* was estimated for horses as a Pert distribution based on data from WAHID and FAO used by de Vos *et al*. [1]: Pert(4x10^-6^, 5.02x10^-4^, 1x10^-3^). For donkeys and zebras, *CI_ijm_* in high risk region were respectively assumed equal at 1.2x10^-2^ and 1.6x10^-2^ based on rate of seroconversion in foals, the surviving foaling rate and the offspring rate [15] [16].

***r_jm_* = Prevalence of infected vectors during an outbreak in the region *j***

We applied for the prevalence of infected vector the same process than for infected host. Thus *r_jm_* is considered as a constant in all region *j*. In high risk region *r_jm_* is assumed at 0.014 based on data from South Africa [17]. In low risk region, as for equidae the number of infectious animal is divided by 10^-2^ between high risk regions and low risk regions, the rate was here estimated as 1.4x10^-4^.

**BIBLIOGRAPHIE**

1. De Vos CJ, Hoek CA, Nodelijk G: **Risk of introducing African horse sickness virus into the Netherlands by international equine movements**. *Prev Vet Med* 2012, **106**:108–122.

2. Kazeem MM, Rufai N, Ogunsan EA, Lombin LH, Enurah LU, Owolodun O: **Clinicopathological Features Associated with the Outbreak of African Horse Sickness in Lagos, Nigeria**. *J Equine Vet Sci* 2008, **28**:594–597.

3. Backer JA, Nodelijk G: **Transmission and Control of African Horse Sickness in The Netherlands: A Model Analysis**. *PLoS ONE* 2011, **6**.

4. Wittmann EJ, Mellor PS, Baylis M: **Effect of temperature on the transmission of orbiviruses by the biting midge, Culicoides sonorensis**. *Med Vet Entomol* 2002, **16**:147–156.

5. Lo Iacono G, Robin CA, Newton JR, Gubbins S, Wood JLN: **Where are the horses? With the sheep or cows? Uncertain host location, vector-feeding preferences and the risk of African horse sickness transmission in Great Britain**. *J R Soc Interface* 2013, **10**:20130194–20130194.

6. Venter GJ, Wright IM, Paweska JT: **A comparison of the susceptibility of the biting midge Culicoides imicola to infection with recent and historical isolates of African horse sickness virus**. *Med Vet Entomol* 2010, **24**:324–328.

7. Baylis M, O’Connell L, Mellor PS: **Rates of bluetongue virus transmission between Culicoides sonorensis and sheep**. *Med Vet Entomol* 2008, **22**:228–237.

8. Wilson A, Mellor PS, Szmaragd C, Mertens PPC: **Adaptive strategies of African horse sickness virus to facilitate vector transmission**. *Vet Res* 2009, **40**.

9. Gubbins S, Carpenter S, Baylis M, Wood JL., Mellor PS: **Assessing the risk of bluetongue to UK livestock: uncertainty and sensitivity analyses of a temperature-dependent model for the basic reproduction number**. *J R Soc Interface* 2008, **5**:363–371.

10. Ninio C, Augot D, Delecolle J-C, Dufour B, Depaquit J: **Contribution to the knowledge of Culicoides (Diptera: Ceratopogonidae) host preferences in France**. *Parasitol Res* 2011, **108**:657–663.

11. Viennet E, Garros C, Gardès L, Rakotoarivony I, Allène X, Lancelot R, Crochet D, Moulia C, Baldet T, Balenghien T: **Host preferences of Palaearctic Culicoides biting midges: implications for transmission of orbiviruses**. *Med Vet Entomol* 2013, **27**:255–266.

12. Jones RH, Foster NM: **The effet of repeated blood meals for Bluetongue on the infection rate of Culicoides variipennis**. *J Med Entomol* 1971, **8**:499–501.

13. Rodriguez M, Ladero JL, Castaño M, Hooghuis H: **African horse sickness in Spain: Epizootiological and regulatory consideretions**. *J Equine Vet Sci* 1992, **12**:395–400.

14. Rodriguez M, Hooghuis H, Castaño M: **African horse sickness in Spain**. *Vet Microbiol* 1992, **33**:129–142.

15. Barnard BJ: **Circulation of African horsesickness virus in zebra (Equus burchelli) in the Kruger National Park, South Africa, as measured by the prevalence of type specific antibodies**. *Onderstepoort J Vet Res* 1993, **60**:111–117.

16. Penzhorn BL: **Reproductive characteristics of a free-ranging population of Cape mountain zebra (Equus zebra zebra)**. *J Reprod Fertil* 1985, **73**:51–57.

17. Scheffer EG, Venter GJ, Labuschagne K, Page PC, Mullens BA, MacLachlan NJ, Osterrieder N, Guthrie AJ: **Comparison of two trapping methods for Culicoides biting midges and determination of African horse sickness virus prevalence in midge populations at Onderstepoort, South Africa**. *Vet Parasitol* 2012, **185**:265–273.
