# Supplementary material for: A spatiotemporal model to assess the introduction risk of African horse sickness by import of animals and vectors in France
Source: BMC Vet Res. 2015 Jun 4;11:127. doi: 10.1186/s12917-015-0435-4 (PMC4455332; doi:10.1186/s12917-015-0435-4)
Supplement: Additional file 3: — Model calculation for PW-vector. Details of calculation regarding the AHSV introduction via the import of an infectious vector. [file 12917_2015_435_MOESM3_ESM.docx]

**Additional file 3: Model calculation for PW-vector**

All the parameters used are detailed in the Additional file 2.

The probability to introduce a single vector from *j* to *k* during the month *m* which is able to induce an entire transmission cycle in which at least one local host is infected by a local vector is defined as:

$$P\left( introB_{jkm} \right)=P(relB_{jkm})\times P(estB_{jkm})$$

Where $P\left( \mathrm{rel}B_{\mathrm{jkm}} \right)=P\left( \mathrm{trans}_{\mathrm{culi}_{\mathrm{jm}}} \right)\times P\left( \mathrm{sur}{v_{\mathrm{trans}}}_{\mathrm{jkm}} \right)\times P\left( inf\_\mathrm{culi}_{\mathrm{jm}} \right)\times n_{\mathrm{tran}s_{\mathrm{jkm}}}$

And $P\left( \mathrm{est}B_{\mathrm{jkm}} \right)=P\left( \mathrm{sur}v_{{arrival}_{\mathrm{jkm}}} \right)\times b_{\mathrm{equ}i_{k}}\times I_{\mathrm{VH}}\times\left[ 1-\left[ 1-I_{\mathrm{VH}}\times P\left( \mathrm{surv}_{\mathrm{km}} \right)\times b_{\mathrm{equ}i_{k}}\times I_{\mathrm{HV}} \right]^{\mathrm{culi}_{\mathrm{km}}} \right]$

With *culi_km_* the number of vector feeding on an infected viraemic imported host calculated as:

*culi_km_* = BR_km_ x Vir x C_km_

1. $\mathbf{P}\left( \boldsymbol{inf\_cul}\mathbf{i}_{\mathbf{jm}} \right)\mathbf{=}$**Probability for a vector to be infected the month *m* in area *j***

$$P\left( inf\_culi_{\mathrm{jm}} \right)=PO_{\mathrm{jm}}\times r_{\mathrm{jm}}$$

2. $\mathbf{P}\left( \mathbf{tran}\mathbf{s}_{\mathbf{cul}\mathbf{i}_{\mathbf{jm}}} \right)\mathbf{=}$**Probability for a vector to be transported after infection from area *j***

Only a vector which is infected and transported poses a risk, therefore we only consider those vectors that are infected and transported during their life time. We assume that an infected vector will be infected at a uniformly distributed time during its life, D_inf_. Additionally, we assume that a vector is transported at a uniformly distributed moment during its life time, which is exponentially distributed with mean 1/MR_jm_. The probability that the moment of transportation occurs after the infection event is equal to the part of the total lifetime of the vector that it is infected. Thus $P\left( \mathrm{tran}s_{\mathrm{cul}i_{\mathrm{jm}}} \right)$ is estimated, as made by Napp et al. [1], as:

$$P\left( \mathrm{trans}_{\mathrm{culi}_{\mathrm{jm}}} \right)=\frac{\left( {1/MR}_{\mathrm{jm}}-D_{\inf} \right)}{{1/MR}_{\mathrm{jm}}}=1-D_{\inf}\mathrm{MR}_{\mathrm{jm}}$$

NB: Temperature in departure area *j* was assumed to be constant over months and thus MR_jm_ is here also constant over months.

1. $\mathbf{P}\left( \mathbf{sur}\mathbf{v}_{\mathbf{tran}\mathbf{s}_{\mathbf{jkm}}} \right)\mathbf{=}$**Probability for a vector to stay alive from *j* until the arrival in area *k* during the month *m***

The conditions during travel (e.g. temperature) are assumed to not affect the viability of culicoides except when pest control is applied (worst case scenario). There is no data available on survival rate of culicoides in an unfavorable context as assumed to occur during transport. Moreover the conditions during transports have a high variability and information are impossible to collect.

The probability to stay alive until the arrival is the probability to survive until transport and during the time of transport.

$$P\left( \mathrm{sur}v_{\mathrm{tran}s_{\mathrm{jkm}}} \right)=e^{-\mathrm{MR}_{\mathrm{jm}}\times(D_{\mathrm{trans}}+t_{\mathrm{jk}})}\times(1-Prot_{\mathrm{vect}})$$

1. $\mathbf{P}\left( \mathbf{sur}\mathbf{v}_{\mathbf{arriva}\mathbf{l}_{\mathbf{jkm}}} \right)\mathbf{=}$**The vector survives to the transport from *j*, the EIP and can have at least a blood meal after the end of EIP and when arrives in the area *k* the month *m***

If T_B_ < 0 culicoides are assumed to not survive

= 0

If (N_m_.GC_jm_) > (D_transp_ + t_jk_)

$$=e^{\mathrm{MR}_{\mathrm{km}}\times\left( D_{\mathrm{trans}}+t_{\mathrm{jk}} \right)-N_{m}\times GC_{\mathrm{jm}}}$$

If (N_m_.GC_jm_) < (D_transp_ + t_jk_)

If t_jk_ > GC_jm_

we assume that the last GC_m_ is spent half during transport and half in the arrival area *k.*

$$=e^{-\mathrm{MR}_{\mathrm{km}}\times\frac{GC_{\mathrm{jm}}}{2}}$$

If t_jk_ < GC_jm_

we assume that the last GC_m_ is spent half in the departure area *j* and half in the arrival area *k*.

$$=e^{-\mathrm{MR}_{\mathrm{km}}\times\frac{GC_{\mathrm{jm}}-t_{jk}}{2}}$$

NB : if T_k_ < T_min (9.5°C), where T_min is the minimal temperature for formulae for MR and GC (if T_k_ is lower, the formulae are not valid), we will use the T_min in our calculus (worst case scenario).

1. $\mathbf{P}\left( \mathbf{sur}\mathbf{v}_{\mathbf{km}} \right)\mathbf{=}$**Probability that the local vector survives to the EIP and can have a blood meal during the month *m* in the area *k***

$$P\left( \mathrm{sur}v_{\mathrm{km}} \right)= e^{-(N_{km}\times GC_{km}\times MR_{km})}$$

**BIBLIOGRAPHIE**

1. Napp S, García-Bocanegra I, Pagès N, Allepuz A, Alba A, Casal J: **Assessment of the risk of a bluetongue outbreak in Europe caused by Culicoides midges introduced through intracontinental transport and trade networks.** *Med Vet Entomol* 2012, 27:19–28.
